# Effectiveness and waning of protection with the BNT162b2 vaccine against the SARS-CoV-2 Delta variant in immunocompromised individuals

**DOI:** 10.3389/fimmu.2023.1247129

**Published:** 2023-11-02

**Authors:** Zoltán Szekanecz, Zoltán Vokó, Orsolya Surján, Éva Rákóczi, Szilvia Szamosi, Gabriella Szűcs, Éva Szekanecz, Cecília Müller, Zoltán Kiss

**Affiliations:** ^1^ Department of Rheumatology, Faculty of Medicine, University of Debrecen, Debrecen, Hungary; ^2^ Center for Health Technology Assessment, Semmelweis University, Budapest, Hungary; ^3^ Syreon Research Institute, Budapest, Hungary; ^4^ Department of Deputy Chief Medical Officer II., National Public Health Center Management, Budapest, Hungary; ^5^ Department of Oncology, Faculty of Medicine, University of Debrecen, Debrecen, Hungary; ^6^ Department of Chief Medical Officer, National Public Health Center Management, Budapest, Hungary; ^7^ Second Department of Medicine and Nephrology-Diabetes Center, University of Pécs Medical School, Pécs, Hungary

**Keywords:** SARS-CoV-2, COVID-19, immunocompromised patients, BNT162b2 vaccine, booster, vaccine effectiveness

## Abstract

**Introduction:**

In Hungary, the HUN-VE 3 study determined the comparative effectiveness of various primary and booster vaccination strategies during the Delta COVID-19 wave. That study included more than 8 million 18-100-year-old individuals from the beginning of the pandemic. Immunocompromised (IC) individuals have increased risk for COVID-19 and disease course might be more severe in them. In this study, we wished to estimate the risk of SARS-CoV-2 infection and COVID-19 related death in IC individuals compared to healthy ones and the effectiveness of the BNT162b2 vaccine by reassessing HUN-VE 3 data.

**Patients and methods:**

Among the 8,087,988 individuals undergoing follow-up from the onset of the pandemic in the HUN-VE 3 cohort, we selected all the 263,116 patients with a diagnosis corresponding with IC and 6,128,518 controls from the second wave, before vaccinations started. The IC state was defined as two occurrences of corresponding ICD-10 codes in outpatient or inpatient claims data since 1 January, 2013. The control group included patients without chronic diseases. The data about vaccination, SARS-CoV-2 infection and COVID-19 related death were obtained from the National Public Health Center (NPHC) during the Delta wave. Cases of SARS-CoV-2 infection were reported on a daily basis using a centralized system via the National Public Health Center (NPHC).

**Results:**

Out of the 263,116 IC patients 12,055 patients (4.58%) and out of the 6,128,518 healthy controls 202,163 (3.30%) acquired SARS-CoV-2 infection. Altogether 436 IC patients and 2141 healthy controls died in relation to COVID-19. The crude incidence rate ratio (IRR) of SARS-CoV-2 infection was 1.40 (95%CI: 1.37-1.42) comparing IC patients to healthy controls. The crude mortality rate ratio was 4.75 (95%CI: 4.28-5.27). With respect to SARS-CoV-2 infection, interestingly, the BNT162b2 vaccine was more effective in IC patients compared to controls. Primary vaccine effectiveness (VE) was higher in IC patients compared to controls and the booster restored VE after waning. VE regarding COVID-19 related death was less in IC patients compared to healthy individuals. Booster vaccination increased VE against COVID-19-related death in both IC patients and healthy controls.

**Conclusion:**

There is increased risk of SARS-CoV-2 infection and COVID-19 related mortality in IC patient. Moreover, booster vaccination using BNT162b2 might restore impaired VE in these individuals.

## Introduction

Since the outbreak of the Coronavirus Disease-19 (COVID-19) pandemic, we have learned a lot about the evolution, clinical picture, diagnosis, prevention, treatment and outcome of the disease (1). Vaccination is the most effective method against SARS-CoV-2, as well as any other viruses. Vaccination, in most cases, triggers sufficient immune response to prevent severe COVID-19 (1–3).

Hungary registered more than 1.2 million SARS-CoV-2 infections and more than 39,000 COVID-19 related deaths by the end of 2021. Out of these cases, 450,000 infections and 9,000 deaths were associated with the Delta (B.1.617.2) variant of SARS-CoV-2 (4–6). In the nationwide HUN-VE 1 study, we reported high or very high short-term effectiveness of five COVID-19 vaccines against the Alpha variant of SARS-CoV-2, as well as COVID-19-related mortality (4). In order to maintain protection against the emerging new Delta and Omicron variants, most European countries including Hungary started booster vaccinations in mid-2021 (5, 6). In the HUN-VE 2 and HUN-VE 3 studies we were able to evaluate the comparative effectiveness of various booster vaccination strategies and to assess the durability of protection provided by primary and booster immunization protocols during the same COVID-19 wave (5, 6). The nationwide HUN-VE 2 study confirmed the efficacy of single and double booster vaccination strategies against the Delta and Omicron variants, as well as COVID-19-associated death (5).

This was followed by the HUN-VE 3 study, which included more than 8 million 18-100- year-old individuals from the beginning of the pandemic. This study confirmed the outstanding benefit of booster vaccinations using the BNT162b2 and mRNA-1273 mRNA, as well as Ad26.COV2.S vector vaccines in comparison to the ChAdOx1, GAM-COVID-Vac and BBIBP-CorV vaccines during the Delta wave. This study also demonstrated waning of efficacy with all vaccine types. The major strength of the HUN-VE 3 study was that this was the first comparative study on the efficacy of six different vaccine types during the Delta wave (6).

Immunocompromised (IC) individuals including patients with autoimmune diseases, immunodeficiencies, hematologic malignancies, transplanted patients and those receiving immunosuppressive drugs, primarily corticosteroids, exert increased risk for various infections including SARS-CoV-2. Moreover, the course of COVID-19 might be more severe in these individuals (1, 7–13). In addition, vaccines might have diminished efficacy and their immunogenicity might wane over time in these patients (9, 10, 14–16). For example, in a recent systematic review and meta-analysis carried out on 3207 IC patients and 1726 healthy individuals receiving two doses of mRNA vaccine, seroconversion was 48% lower in IC individuals compared to controls. Transplant recipients had much lower rates of seroconversion compared to patients with autoimmune conditions or malignancies (9). Although the efficacy of the 4^th^ vaccine dose might be somewhat lower in IC, especially transplanted patients (17, 18), the 4^th^ dose was associated with improved seroconversion and antibody titre levels (19). According to data from the COVAX registry, the safety profile of SARS-CoV-2 vaccines in IC patients was reassuring and comparable with non-IC subjects (20).

The aim of this investigation was to determine the risk of SARS-CoV-2 infection and COVID-19 related death in IC individuals compared to healthy ones during the second wave and the effectiveness of BNT162b2 vaccine in IC patients by reanalysing data emerging from the HUN-VE 3 study among them during the Delta wave (6). This is the most robust analysis of SARS-CoV-2 vaccine efficacy in Hungarian IC patients. In addition, we also evaluated the durability of protection after the administration of various vaccine combinations in order to provide recommendations for the vaccination strategy of IC patients.

## Patients and methods

### Study population and definitions

The study population included Hungarian residents aged 18 to 84 years in an IC state and a healthy control group of non-IC cases. Among the 8,087,988 individuals undergoing follow-up from the onset of the pandemic in the HUN-VE 3 cohort (6), we selected all the 263,116 patients with a diagnosis corresponding with IC and 6,128,518 controls from the second wave between 4.9.2020 and 25.12.2020 (**Table 1**). The IC state was defined as two occurrences of corresponding ICD-10 codes in outpatient or inpatient claims data since 1 January, 2013, using the second occurrence as the date of diagnosis (**Table S1**). We included patients with immuno-haematological, autoimmune thyroid, neurological, renal, hepatic, pulmonary diseases, as well as Raynaud’s syndrome, inflammatory bowel diseases, psoriasis and other autoimmune skin diseases, rheumatoid arthritis, spondyloarthritis, autoimmune connective tissue diseases, as well as transplant patients. As this study was performed on individuals during the second wave, none of these subjects were vaccinated.

**Table 1:** Study populations and occurrence of SARS-CoV-2 infection and COVID-19-related death during the Delta wave of the pandemic in immunocompromised (IC) patients compared to healthy individuals aged 18-84 years.

age (years)	N		COVID-19 cases		Death	
	healthy	IC	healthy	IC	healthy	IC
**18-24**	663,771	16,660	19,435	691	6	1
**25-34**	1,181,193	29,748	39,073	1,433	32	2
**35-44**	1,408,743	43,427	53,988	2,571	54	8
**45-54**	1,234,872	46,016	50,166	2,871	167	28
**55-64**	817,970	50,451	24,580	2,200	386	89
**65-74**	582,871	52,027	9,674	1,440	710	165
**75-84**	239,098	24,787	5,247	849	786	143
**Total**	6,128,518	263,116	202,163	12,055	2,141	436

The control group included patients without chronic diseases. The presence of chronic diseases was identified based on in- and outpatient health service utilization and prescription data from the National Health Insurance Fund. The following chronic conditions were considered: cardiovascular diseases (myocardial infarction, angina, chronic heart failure, peripheral vascular disease, and stroke), diabetes mellitus (type 1 and type 2), immunosuppression (immunosuppressive therapy and transplantation), chronic pulmonary diseases (asthma bronchiale and chronic obstructive pulmonary diseases), neoplasms, and chronic kidney diseases. The definitions of chronic diseases are presented in **Table S1**.

The data about vaccination, SARS-CoV-2 infection and COVID-19 related death were registered and provided by the National Public Health Center (NPHC). Cases of SARS-CoV-2 infection were reported on a daily basis using a centralized system via the (NPHC). The report was based on (i) COVID-19-related symptoms identified by hospital physicians and general practitioners, (ii) positive nucleic acid amplification test reported by microbiological laboratories. Cases identified by symptoms were confirmed by PCR or antigen test included in the EC rapid test list (21). COVID-19 related mortality was defined as death during SARS-CoV-2 positivity, regardless of whether death was the direct consequence of COVID-19 infection or other underlying causes. Patients with confirmed SARS-CoV-2 infection who died without previously declared recovery and another clear cause of death were classified as COVID-19 related deaths. The definition was based on WHO recommendations and defined by the healthcare government (22).

Individuals with history of SARS-CoV-2 infection prior to 4 September, 2020 were excluded from the study the risk of infection, while the mortality outcome studies could include subjects who had SARS-CoV-2 infection beforehand.

Age groups were split into the following categories 18-24, 25-34, 35-44, 45-54, 55-64, 65-74 and 75-84 years (**Table 1**).

The analysis of vaccine effectiveness (VE) was conducted during the Delta wave between 8.11.2021 and 31.12.2021. We only used the data of individuals vaccinated by the BNT162b2 (Pfizer-BioNTech) vaccine as most other vaccines have not been recommended to IC patients in Hungary, and on the other hand the number of those who received other vaccines were too few to be involved in the analysis.

The study was approved by the Central Ethical Committee of Hungary (OGYÉI/10296-1/2022 and IV/1722- 1/2022/EKU).

### Statistical analysis

First, we used the Kaplan-Meier method to estimate the risk of SARS-CoV-2 infection and COVID-19 related mortality over time during the second wave of the pandemic in Hungary. The period considered was from 4 September 2020 till 25 December 2020. We compared immunosuppressed individuals with healthy ones, the latter being defined as those without history of chronic diseases since 1 January 2013.

We calculated the crude incidence rates of the outcomes as number of events divided with person-time of observation by study group. We then calculated incidence rate ratios as crude effect measure.

Next, we employed Royston-Parmar models with restrictive cubic spline, incorporating three internal knots placed at the 25th, 50th and 75th centiles of the distribution of the uncensored log survival times. We estimated hazard ratios (HR), adjusted for age, sex. A decision to represent age as either categorical or continuous variable in the models made through likelihood ratio test and Akaike information criterion. As a result, age was treated as a categorical variable in the model estimating the hazard of infection, and as a continuous variable in the model for death. Additionally, we studied the interaction with age using interaction terms and likelihood ratio test, and the time dependence of the hazard ratio comparing the two study groups.

During the Delta wave, which occurred between 13 September, 2021 and 31 December, 2021 we estimated VE defined as one minus the hazard ratio. We used mixed effect negative binomial regression models by study groups (i.e., immunosuppressed and healthy individuals) to derive adjusted incidence rate ratios (IRRs) with 95% CIs for each outcome. These were adjusted for age, sex, history of different chronic diseases, and calendar day modelled as a random effect. The models were random intercept models, which allowed for varying incidence rates in the reference category (i.e., unvaccinated) each day but presumed a fixed effect of the intervention. We examined vaccination categories by time elapsed since primary vaccination (14-120 days, 121-180 days, more than 180 days). We also included those who received a booster vaccination at least 14 days earlier.

## Results

### Study population characteristics, SARS-CoV-2 infections and COVID-19 related mortality during the 2^nd^ wave

The age distribution of the study population is shown in **Table 1**. Of the 263,116 IC patients 12,055 patients (4.58%) and of the 6,128,518 healthy controls 202,163 (3.30%) acquired SARS-CoV-2 infection, and 436 IC patients and 2141 healthy controls died related to COVID-19 (**Table 1**).

**Figure 1:**
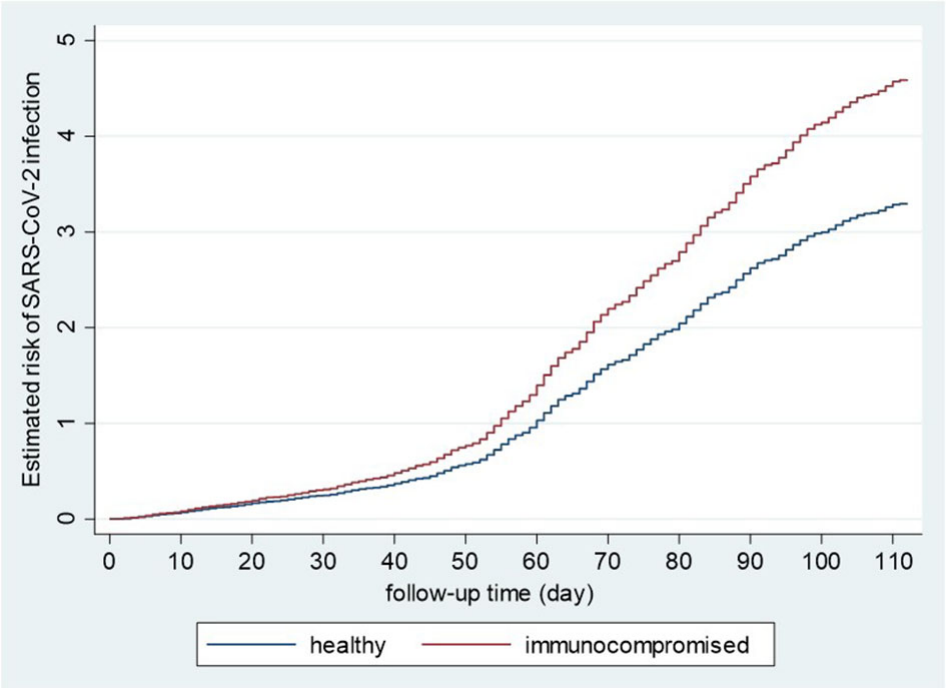
Kaplan-Meier curves of the risk of SARS-CoV-2 infection in immunocompromised and healthy controls. Healthy and immunocompromised, unvaccinated populations, including men and women aged 18-84, were analyzed using the Kaplan-Meier method for the estimated risk of SARS-CoV-2 infection during the second wave.

The incidence rate (IR) of SARS-CoV-2 infection among IC patients and healthy controls were 41.7 (95%CI: 41.0-42.5) and 29.9 (95%CI: 29.7-30.0) per 100,000 person-days, respectively. The crude incidence rate ratio (IRR) was 1.40 (95%CI: 1.37-1.42) comparing IC patients to healthy controls (**Figure 1**).

**Figure 2:**
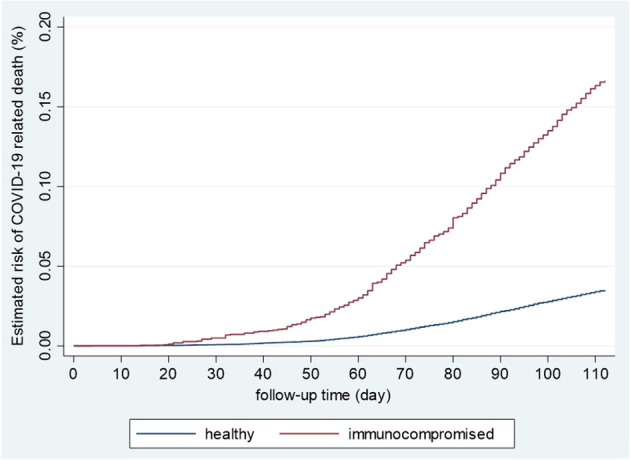
Kaplan-Meier curves of the risk of COVID-19-related death in immunocompromised and healthy controls. Healthy and immunocompromised, unvaccinated populations, including men and women aged 18-84, were analyzed using the Kaplan-Meier method for the estimated risk of COVID-19á-related death during the second wave.

The mortality rate among IC patients and healthy controls were 1.48 (95%CI: 1.35-1.63) and 0.31 (95%CI: 0.30-0.33) per 100,000 person-days, respectively. The crude mortality rate ratio was 4.75 (95%CI: 4.28-5.27) compared to healthy controls (**Figure 2**).

**Figure 3:**
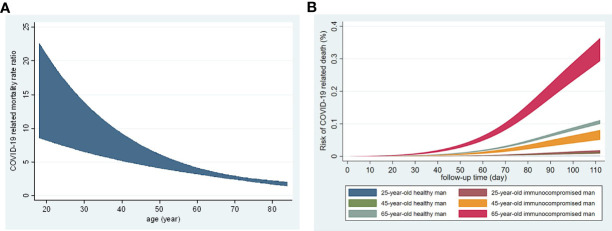
Age-dependence of COVID-19-related death in unvaccinated immunocompromised patients and healthy indiviaduals during the second wave. **(A)** Rate ratio of COVID-19-related mortality by age adjusted for sex comparing immunocompromised patients and healthy individuals (relative risk). **(B)** Estimated risk of COVID-19-related death by age in men (absolute risk).

The risk of SARS-CoV-2 infection increased in IC patients compered to controls (**Table 2**). The hazard ratio (HR) varied between 1.38 and 1.65 by age groups. The largest excess risk was observed among IC patients at the of 65-74 years (HR: 1.65) and 75-84 years (HR: 1.56). **Figures 3A, B** reflect two aspects of the increased risk of COVID-19-related mortality in IC patients compared to non-IC controls: increased relative and absolute risk. **Figure 3A** shows the relative rate of COVID-19-related death as a function of time comparing IC patients to controls. Indeed, the excess risk of COVID-19 related death decreased with age, showing very large difference on the ratio scale in young persons and very little among elderly (**Figure 3A**). To illustrate the absolute effect of this interaction with age, we estimated the risk of death for men at the age of 25, 45 and 65 years (**Figures 3B**). The highest mortality was observed in 65-year-old IC men. Their mortality was much higher than 65-year-old healthy men. Interestingly, the mortality of 45-year-old IC men was similar to 65-year-old healthy individuals. The mortality of 25-year-old IC, as well as 25- and 45-year-old healthy men was very low and similar to each other (**Figure 3B**).

**Table 2:** Hazard ratio of SARS-CoV-2 infection adjusted for sex by age comparing immunocompromised patients to healthy individuals aged 18-84 years.

Age (years)	HR (95% CI)
**18-24**	1.38 (1.28-1.49)
**25-34**	1.42 (1.35-1.50)
**35-44**	1.51 (1.45-1.57)
**45-54**	1.50 (1.45-1.56)
**55-64**	1.41 (1.35-1.48)
**65-74**	1.65 (1.56-1.74)
**75-84**	1.56 (1.45-1.68)

### Assessment of vaccine effectiveness and waning

**Figure 4:**
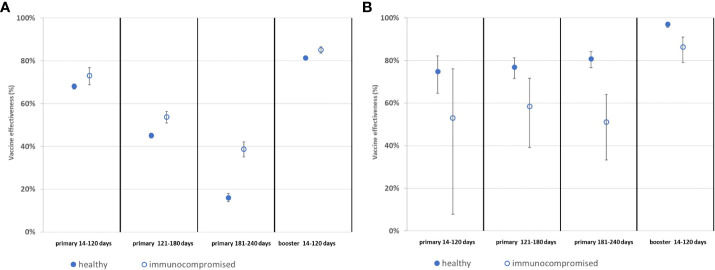
Effectiveness of the BNT162b2 vaccine during the Delta wave of COVID-19 pandemic in immunocompromised patients compared to the healthy population. **(A)** Effectiveness of BNT162b2 vaccination against SARS-CoV-2 infection. **(B)** Effectiveness of BNT162b2 vaccination against COVID-19-related death.

With respect to SARS-CoV-2 infection, interestingly, the BNT162b2 vaccine was more effective in IC patients compared to controls (**Figure 4A**, **Tables S2, S3**). When comparing VE against SARS-CoV-2 infection in the three time periods post-primary vaccination shown above, adjusted VE decreased from 68.1% to 16.1% in healthy controls (**Table S2**) and from 73.0% to 38.7% in IC patients (**Table S3**) suggesting anti-infection VE waning in both study groups, which was more pronounced in healthy individuals (**Figure 4A**). Primary VE was higher in IC patients compared to controls throughout the observation period (**Figure 4A**). Booster vaccination resulted in 81.3-85.1% adjusted anti-infection VE (**Tables S2, S3**, **Figure 4A**) indicating that the booster restores VE after waning.

VE regarding COVID-19 related death was less in IC patients compared to healthy individuals (**Figure 4B**, **Tables S4, S5**). This was observed irrespective of the time elapsed from vaccination after primary, as well as 14 to 120 days after booster vaccination. Again, when comparing VE against COVID-19-related death in the three time periods post-primary vaccination, adjusted VE stayed between 74.9% and 80.8% in healthy controls (**Table S4**) and also between 51.1% and 58.4% in IC patients (**Table S5**) suggesting that there is no anti-death VE waning in any of the two groups (**Figure 4B**). Booster vaccination resulted in 97.0% versus 86.3% adjusted VE (**Tables S4, S5**) indicating that the booster vaccination increases VE against COVID-19-related death in both IC patients and healthy controls. Yet, booster VE was also lower in IC patients in comparison to controls (**Figure 4B**, **Tables S4, S5**).

## Discussion

HUN-VE 2 and 3 were the largest Hungarian cohort that assessed SARS-CoV-2 infection rate and COVID-19-related mortality, as well as the effectiveness of various anti-SARS-CoV-2 vaccines on these two clinical outcomes (5, 6). Here we assessed the same outcome parameters in more than 263,000 Hungarian IC patients in comparison to more than 6 million healthy individuals. The risk of SARS-CoV-2 infection and COVID-19-related mortality was higher in IC patients compared to controls. The highest infection risks were observed in subjects with the age of 65 years or more. In relative terms IC increased the risk of COVID-19 related mortality in young individuals. In addition, VE against SARS-CoV-2 infection was higher, while that against COVID-19-related death was lower among IC patients compared to healthy controls. We observed waning of primary VE against SARS-CoV-2 infection in both IC patients and controls, however, waning was more pronounced in healthy individuals. We did not observe waning of primary VE against COVID-19-related mortality in any of the study groups. Booster vaccination restored/increased VE against both SARS-CoV-2 infection and COVID-19-associated mortality.

Some other investigators reported increased risk of SARS-CoV-2 infection among patients with various autoimmune diseases and immunodeficiencies (7, 11, 23). In a meta-analysis, corticosteroids, conventional synthetic disease-modifying drugs (csDMARD) and combination therapy using either biological (bDMARD) or targeted synthetic DMARDs (tsDMARD) along with csDMARDs increased the risk of COVID-19-related hospitalization and death. On the other hand, b/tsDMARD monotherapy decreased the risk of these outcomes (7).

In IC patients compared to healthy controls, VE against SARS-CoV-2 infection was higher, while that against COVID-19-related death was lower. There have been relatively few studies on VE in IC patients (13, 24). In most studies, immunogenicity of vaccination has been assessed and, in general, IC patients exerted decreased humoral responses to anti-SARS-CoV-2 vaccines compared to non-IC individuals (10, 15, 16, 25–27). For example, Furer et al. (15) reported impaired immunogenicity of the BNT162b2 vaccine in various autoimmune diseases including inflammatory myopathies and ANCA-associated vasculitides. In the OCTAVE study, Kearns et al. (16) reported that overall, 60% of IC patients had sufficient vaccine immunogenicity, but 11% of them failed to generate any antibodies. We have recently compared the immunogenicity of five anti-SARS-CoV-2 vaccines available in Hungary in autoimmune-rheumatic patients and healthy volunteers. Anti-RBD IgG levels were slightly lower in rheumatic patients compared to controls. In addition, the two mRNA vaccines yielded the best immunogenicity in both patients and controls (10). In solid organ transplanted patients, low anti-SARS-CoV IgG titers and low number of reactive IFNγ-producing cells were observed after three doses of BNT162b2 (28). Lee et al. (25) published a meta-analysis of the efficacy of SARS-CoV-2 vaccines in IC patients. Seroconversion rates were significantly lower in IC patients, especially organ transplant recipients (25). Finally, Kreuzberger et al. (26) conducted a Cochrane Systematic Review on immunity after SARS-CoV-2 vaccination in IC patients. This review included 318 studies with a total of more than 5 million participants including patients with haematological and solid malignancies, kidney transplant and autoimmune rheumatic diseases, as well as pregnant or breastfeeding women. Seven vaccines were investigated. Again, the majority of studies focused on immunogenicity outcomes and adverse events (26).

Immunosuppressive drugs administered to patients with autoimmune diseases could attenuate the immunogenicity of the basic regimen, as well as the 3^rd^ and 4^th^ booster doses (10, 14, 15, 17, 29, 30). Among these agents, primarily B-cell blocking biologics, such as rituximab, as well as corticosteroids, mycophenolate mofetil and possibly JAK inhibitors might interfere with vaccine immunogenicity including both antibody production and seroconversion, therefore booster doses are recommended (10, 14, 17, 29–31). On the other hand, with the exception of rituximab, immunosuppressants did not increase the risk of mechanical ventilation and in-hospital death in IC patients (32). Wieske et al. (33) conducted a multicentre study on more than 3000 participants, both IC patients and controls in the Netherlands. B-cell depleting therapy, sphingosine 1-phosphate receptor (S1P) modulators and mycophenolate mofetil (MMF) combined with corticosteroids were associated with impaired seroconversion following standard vaccination. A 3^rd^ booster dose increased seroconversion in patients receiving MMF, but not in those treated with B-cell inhibitors or S1P modulators. Simon et al. (34) suggested that in IC patients, the reduced and/or delayed responses to anti-SARS-CoV-2 vaccination is dependent primarily on the underlying disease rather than concomitant treatment.

There have been very few hard-endpoint VE studies in IC patients. Di Fusco et al. (24) performed a targeted literature review on real-worlds studies and published an expert opinion. VE of the widely available four COVID-19 vaccines was between 64-90% against SARS-CoV-2 infection and 63-100% against COVID-19-related hospitalization among the fully vaccinated IC populations. VE in the studied IC populations was lower than in the general population (24). Papagoras et al. (13) conducted a study in Greece. In that cohort of IC patients with autoimmune rheumatic diseases, both hospitalisation (29.3% vs 10.3%) and mortality (4.1% vs 0%) rates were higher in unvaccinated compared to fully vaccinated patients (13). In our present study, VE against SARS-CoV-2 infection was higher, while that against COVID-19 related death was lower in IC patients compared to controls. We postulate that IC patients exerted different behaviour compared to non-IC controls with respect to medical care during the pandemic. IC patients have been very closely controlled and their vaccination strategy might have been different in comparison to controls. This might explain the somewhat unexpected results regarding VE against SARS-CoV-2 infection. Certainly, there might be other reasons for these observations. On the other hand, mortality might be a lot more reliable hard endpoint in this respect. Therefore, our results suggesting impaired VE against COVID-19-related death in IC patients compared to controls should be considered more seriously.

We observed waning of primary VE against SARS-CoV-2 infection but not against COVID-19-associated mortality. Booster vaccination restored VE against SARS-CoV-2 infection and increased VE against mortality. The European Centre for Disease Prevention and Control (ECDC) together with the European Medicines Agency (EMA) recommended additional booster doses (4^th^ dose) to the elderly (>80 years), but also to IC individuals above the age of 60 (35). Again, very little information is available on the VE of boosters applying hard endpoints, such as infection, hospitalization or death. Bonelli et al. (36) found that either homologous or heterologous booster vaccination enhanced immunogenicity in non-seroconverted patients who underwent rituximab treatment. In transplant patients, anti-SARS-CoV-2 IgG could be boosted by the 4^th^ dose of BNT162b2 (28). Schmiedeberg et al. (37) reported the effectiveness of the third booster dose of BNT162b2 in rheumatoid patients with absent or minimal serological response after two doses. In kidney transplant patients, anti-S protein IgG levels after the 3^rd^ booster dose could be further enhanced by administering a 4^th^ vaccine dose (18). We have studied patients with common variable immunodeficiency (CVID), who received two doses of ChAdOx1 first. Basic immunization resulted in low levels of anti-SARS-CoV-2 IgG and T-cell responses. Heterologous BNT162b2 booster administration increased both humoral and cellular immune responses (38). In our cohort, booster vaccination of elderly people (≥ 65 years of age) hospitalized for severe COVID-19 caused by the SARS-CoV-2 Delta variant decreased 28-day all-cause mortality (23).

The major strength of this study is its nationwide, comprehensive nature and its very large sample size. In addition, most other studies only assessed seroconversion and humoral responses but not VE including SARS-CoV-2 infections or mortality. Additionally, few studies could adjust for history of chronic disease when assessing VE. Possible limitations might be due to the real-life data-based retrospective nature of our study. These limitations might include unknown confounding factors and data quality.

VE waning might be an important measure when planning revaccinations in the future based on previous practices (e.g. seasonal flu) (39). In addition, the results and conclusions of the present study could be used by public health specialist to prioritize high-risk populations, such as IC patients SARS-CoV-2, in vaccine distribution.

## Data availability statement

The raw data supporting the conclusions of this article will be made available by the authors, without undue reservation.

## Ethics statement

The studies involving humans were approved by the Central Ethical Committee of Hungary (OGYÉI/10296-1/2022 and IV/1722- 1/2022/EKU). The studies were conducted in accordance with the local legislation and institutional requirements. The ethics committee/institutional review board waived the requirement of written informed consent for participation from the participants or the participants’ legal guardians/next of kin because We only used data from registry, no direct human experiments were performed.

## Author contributions

ZS: conceptualisation, data analysis, draft and final manuscript, supervision. ZV: data collection, data analysis, statistics, interpretation, manuscript drafting and finalisation.OS, ÉR, SS, GS, ÉS, CM: data interpretation, manuscript drafting and finalization. ZK: study coordinator, conceptualisation, data interpretation, manuscript finalization. All authors contributed to the article and approved the submitted version.
