# Supplementary material for: Effectiveness and waning of protection with the BNT162b2 vaccine against the SARS-CoV-2 Delta variant in immunocompromised individuals
Source: Front Immunol. 2023 Nov 2;14:1247129. doi: 10.3389/fimmu.2023.1247129 (PMC10652789; doi:10.3389/fimmu.2023.1247129)
Supplement: Supplementary file 1 [file Table_1.docx]

**Supplementary Table 1.** Definition of history of chronic diseases causing immunocompromised state included in the analysis according to the International Céassification of Diseases (ICD)

| **Disease** | **ICD codes*** |
| --- | --- |
| Kidney diseases | N03, N04 |
| Transplantation | All patients from National Transplantation Registry |
| Autoimmune conditions and immunosuppression | D5900, D5910, D6920, D8690, E05, E0630, G61, G70, I73, K50, K51, K52, K7430, K90, L10, L40, L9590, M0690, M30, M31, M32, M33, M35, M36 |

* Two occurrences of ICD-10 codes in outpatient or inpatient claims data since 1 January 2013
