# Supplementary material for: Effectiveness and waning of protection with the BNT162b2 vaccine against the SARS-CoV-2 Delta variant in immunocompromised individuals
Source: Front Immunol. 2023 Nov 2;14:1247129. doi: 10.3389/fimmu.2023.1247129 (PMC10652789; doi:10.3389/fimmu.2023.1247129)
Supplement: Supplementary file 2 [file Table_2.docx]

**Supplementary Table S2.** Incidence, crude and adjusted effectiveness of vaccine combinations against SARS-CoV-2 infection in the 18-84 years old Hungarian healthy population

| **Vaccination** | **Number of cases** | **Average population size (1000 persons)** | **Incidence rate  (per 100 000 person-days) (95% CI)** | **Crude vaccine efficacy (%) (95% CI)** | **Adjusted vaccine efficacy (%) (95% CI)** |
| --- | --- | --- | --- | --- | --- |
| **Unvaccinated** | 135,468 | 1,958.39 | 62.88 (62.55-63.22) | reference | reference |
| **BNT162b2-vaccinated** |  |  |  |  |  |
| **primary; 14-120 days** | 3,198 | 267.54 | 10.87 (10.49-11.25) | 82.7 (82.1-83.3) | 68.1 (66.8-69.3) |
| **primary; 121-180 days** | 21,607 | 529.84 | 37.07 (36.58-37.57) | 41 (40.2-41.9) | 45.1 (43.9-46.2) |
| **primary; 181-240 days** | 18,435 | 247.36 | 67.75 (66.78-68.74) | -7.7 (-9.4- -6.1) | 16.1 (14.2-17.9) |
| **booster; 14-120 days** | 2,977 | 219.53 | 12.33 (11.89-12.78) | 80.4 (79.7-81.1) | 81.3 (80.6-82.1) |
