# Supplementary material for: Effectiveness and waning of protection with the BNT162b2 vaccine against the SARS-CoV-2 Delta variant in immunocompromised individuals
Source: Front Immunol. 2023 Nov 2;14:1247129. doi: 10.3389/fimmu.2023.1247129 (PMC10652789; doi:10.3389/fimmu.2023.1247129)
Supplement: Supplementary file 3 [file Table_3.docx]

**Supplementary Table S3.** Incidence, crude and adjusted effectiveness of vaccine combinations against registered SARS-CoV-2 infection in the 18-84 years old immunocompromised Hungarian population

| **Vaccination** | **Number of cases** | **Average population size (1000 persons)** | **Incidence rate  (per 100 000 person-days) (95% CI)** | **Crude vaccine efficacy (%) (95% CI)** | **Adjusted vaccine efficacy (%) (95% CI)** |
| --- | --- | --- | --- | --- | --- |
| **Unvaccinated** | 5,169 | 43.63 | 107.69 (104.78-110.67) | reference | reference |
| **BNT162b2-vaccinated** |  |  |  |  |  |
| **primary; 14-120 days** | 187 | 11.34 | 14.99 (12.92-17.30) | 86.1 (83.9-88.0) | 73.0 (68.8-76.7) |
| **primary; 121-180 days** | 1,513 | 33.42 | 41.15 (39.11-43.28) | 61.8 (59.5-63.9) | 53.7 (51.0-56.3) |
| **primary; 181-240 days** | 1,693 | 18.88 | 81.53 (77.69-85.5) | 24.3 (20.0-28.4) | 38.7 (35.1-42.1) |
| **booster; 14-120 days** | 413 | 23.23 | 16.16 (14.64-17.80) | 85.0 (83.4-86.5) | 85.1 (83.5-86.6) |
