# Supplementary material for: Effectiveness and waning of protection with the BNT162b2 vaccine against the SARS-CoV-2 Delta variant in immunocompromised individuals
Source: Front Immunol. 2023 Nov 2;14:1247129. doi: 10.3389/fimmu.2023.1247129 (PMC10652789; doi:10.3389/fimmu.2023.1247129)
Supplement: Supplementary file 4 [file Table_4.docx]

**Supplementary Table S4.** Incidence, crude and adjusted effectiveness of vaccine combinations against COVID-19-related death in the 18-84 years old Hungarian healthy population

| **Vaccination** | **Number of cases** | **Average population size (1000 persons)** | **Incidence rate  (per 100 000 person-days) (95% CI)** | **Crude vaccine efficacy (%) (95% CI)** | **Adjusted vaccine efficacy (%) (95% CI)** |
| --- | --- | --- | --- | --- | --- |
| **Unvaccinated** | 2,354 | 2,519.80 | 0.85 (0.82-0.88) | reference | reference |
| **BNT162b2-vaccinated** |  |  |  |  |  |
| **primary; 14-120 days** | 34 | 270.80 | 0.11 (0.08-0.16) | 86.6 (81.2-90.7) | 74.9 (64.6-82.2) |
| **primary; 121-180 days** | 98 | 538.93 | 0.17 (0.13-0.20) | 80.5 (76.2-84.3) | 76.9 (71.5-81.3) |
| **primary; 181-240 days** | 121 | 253.12 | 0.43 (0.36-0.52) | 48.8 (38.6-57.7) | 80.8 (76.6-84.2) |
| **booster; 14-120 days** | 27 | 220.24 | 0.11 (0.07-0.16) | 86.9 (80.9-91.4) | 97.0 (95.6-98.0) |
