# Supplementary material for: Effectiveness and waning of protection with the BNT162b2 vaccine against the SARS-CoV-2 Delta variant in immunocompromised individuals
Source: Front Immunol. 2023 Nov 2;14:1247129. doi: 10.3389/fimmu.2023.1247129 (PMC10652789; doi:10.3389/fimmu.2023.1247129)
Supplement: Supplementary file 5 [file Table_5.docx]

**Supplementary Table S5.** Incidence, crude and adjusted effectiveness of vaccine combinations against COVID-19-related death in the 18-84 years old immunocompromised Hungarian population

| **Vaccination** | **Number of cases** | **Average population size (1000 persons)** | **Incidence rate  (per 100 000 person-days) (95% CI)** | **Crude vaccine efficacy (%) (95% CI)** | **Adjusted vaccine efficacy (%) (95% CI)** |
| --- | --- | --- | --- | --- | --- |
| **Unvaccinated** | 196 | 71.58 | 2.49 (2.15-2.86) | reference | reference |
| **BNT162b2-vaccinated** |  |  |  |  |  |
| **primary; 14-120 days** | 9 | 11.60 | 0.71 (0.32-1.34) | 71.7 (45.1-87.2) | 53.0 (7.7-76.1) |
| **primary; 121-180 days** | 34 | 34.10 | 0.91 (0.63-1.27) | 63.6 (47.4-47.4) | 58.4 (39.1-71.6) |
| **primary; 181-240 days** | 55 | 19.44 | 2.57 (1.94-3.35) | -3.3 (-40.0-24.8) | 51.1 (33.3-64.1) |
| **booster; 14-120 days** | 26 | 23.34 | 1.01 (0.66-1.48) | 59.3 (38.6-74.1) | 86.3 (79.1-91.0) |
